# Efficacy and safety of sirolimus-eluting stents versus bare-metal stents in coronary artery disease patients with diabetes: a meta-analysis

**DOI:** 10.5830/CVJA-2013-062

**Published:** 2013-10

**Authors:** Yanxiang Qiao, Yuan Bian, Xianliang Yan, Zhenfang Liu, Yuguo Chen

**Affiliations:** Department of Emergency Medicine, Qilu Hospital, Shandong Univeristy, Shandong, China; Department of Emergency Medicine, Qilu Hospital, Shandong Univeristy, Shandong, China; Department of Emergency Medicine, Qilu Hospital, Shandong Univeristy, Shandong, China; Department of Emergency Medicine, Qilu Hospital, Shandong Univeristy, Shandong, China; Department of Emergency Medicine, Qilu Hospital, Shandong Univeristy, Shandong, China

**Keywords:** sirolimus-eluting stent, bare-metal stent, diabetes, meta-analysis, efficacy, safety

## Abstract

**Objective:**

To compare by meta-analysis the efficacy and safety of sirolimus-eluting and bare-metal stents in coronary artery disease (CAD) patients with diabetes.

**Methods:**

PubMed, MEDLINE and EMBASE were searched from 1971 to 2012. Data on the efficacy and safety of sirolimus-eluting and bare-metal stents in patients with diabetes were collected. A meta-analysis was then performed on a total of 1 259 CAD patients with diabetes from six studies. The odds ratio (OR) was used for comparison. Subgroup analysis was performed according to the sample size, year of study, subjects’ geographic area and study method.

**Results:**

Compared with those in the bare-metal stent group (BMS), the subjects in the sirolimus-eluting stent (SES) group had a reduced risk for major cardiac events [OR 0.42, 95% confidence interval (CI): 024–0.74, *p* < 0.01] and target-lesion revascularisation (OR 0.26, 95% CI: 0.11–0.59, *p* < 0.01). There was no difference for myocardial infarction (OR 0.92, 95% CI: 0.61–1.40, *p* > 0.05) or mortality (OR 1.19, 95% CI: 0.74–1.92, *p* > 0.05). Subgroup analysis showed a significant difference for overall risk of major cardiac events between SES and BMS when the sample size was ≤ 90 (OR 0.28, 95% CI: 0.16–0.48, *p* < 0.01), when it was a randomised control trial (RCT) (OR 0.28, 95% CI: 0.19–0.42, *p* < 0.01), or when it was performed on European subjects (OR 0.45, 95% CI: 0.27–0.77, *p* < 0.01). The sensitivity was not different when one study was removed at a time.

**Conclusion:**

Our study confirmed that SES are safer and more effective than BMS in CAD patients with diabetes, as far as major cardiac events are concerned.

## Abstract

According to Nodari *et al.*, compared to patients without diabetes, those with diabetes mellitus (DM) had increased cardiovascular morbidity and mortality, and were more likely to develop congestive heart failure (CHF).^1^ Van Nunen used coronary stents for revascularisation in acute cardiac events and improved the prognosis, with a high success rate and favourable early outcome.^2^

The traditional bare-metal stent (BMS) was initially widely used, with considerable efficacy and safety. However, longterm outcome and restenosis rate has been very discouraging.^3^ Recently, sirolimus-eluting stents (SES) have been increasingly used for treating restenosis after having used BMS, as well as for treating the native coronary narrowing.^4^-^7^

For coronary arterial disease (CAD) patients with diabetes, the outcome, efficacy and safety of SES and BMS remain controversial,^8^-^16^ mainly due to small sample sizes or low statistical power. Meta-analysis, combining results of several studies and producing a single estimate of major events with enhanced precision, has been considered a powerful tool for summarising inconsistent results from different studies.^17^-^20^ Heterogeneity and publication bias can be detected with funnel plots and other methodologies.^21^-^26^

To clarify this controversy, in this study, we performed a meta-analysis and subgroup analysis, along with heterogeneity and publication-bias analysis, and compared the major cardiac events, target-lesion revascularisation, myocardial infarction and mortality rate in CAD patients with diabetes who were treated with SES or BMS.

## Methods

PubMed, MEDLINE, EMBASE, Springer, Elsevier Science Direct, Cochrane Library and Google scholar were searched. The following keywords were used, ‘sirolimus-eluting stents’, ‘baremetal stents’, ‘coronary arterial disease’, ‘diabetes’, ‘diabetic’, ‘safety’, ‘efficacy’, ‘study’ and ‘trial’. The time period was limited from 1 January 1971 to 31 December 2012. The language published in was limited to English only. References of the articles were also checked for additional studies.

Studies included were randomised, controlled trials (RCT) and non-RCT conducted in coronary artery disease patients with diabetes treated with SES or BMS (studies with these two methods compared), regardless of the sample size. Excluded studies were those investigating patients with CAD or DM in only case reports or review articles, duplicated articles, and those with no comparison of SES and BMS.

After the investigators were trained, the data-mining form was developed and modified. The data included study details such as first author, year of study, year of publication, geographical area of subjects, demographics of subjects, and events with follow up after being treated with SES or BMS. According to the standard protocol, two investigators (A and B) mined the data independently, which was reviewed by the third one (C). Discrepancies were resolved through internal and external discussions (with the original investigators).

## Statistical analysis

Analysis was performed with software review manager 5.1 (Cochrane collaboration, http://ims.cochrane.org/revman) and comprehensive meta-analysis (Englewood, NJ); *p* < 0.05 was regarded as statistically significant. Meta-analysis was performed in fixed- or random-effect models.

Odds ratios (OR) and 95% confidence intervals (CI) were estimated in each study. Pooled ORs were obtained using the Mantel-Haenszel method in a fixed-effect model, and the DerSimonian-Laid method in a random-effects model.^24^ The significance of pooled ORs was determined by the *Z*-test. Cochrane’s *Q*-statistic was used to assess within- and betweenstudies variations. A *p* < 0.10 on the Q-statistic was regarded as heterogeneity across the studies. *I*^2^ was also used to test heterogeneity with the formula:

$I^{2}=\;\frac{\left(Q-df\right)}{Q}\;\times 100\%$

where *I*^2^ < 25% means no heterogeneity; *I*^2^ = 25–50% means moderate heterogeneity; *I*^2^ > 50% means large or extreme heterogeneity.^27^

The random-effects model was also used for evaluating the possibility of heterogeneity of studies. Publication bias was evaluated with Egger’s test and funnel plots,^28^ which compensate for each other’s drawbacks. If there is evidence of publication bias, the funnel plot is noticeably asymmetric. For the Egger’s test the significance level was set at 0.05. Sensitivity analysis was also performed to test reliability of the results, by removing one study at a time and repeating the meta-analysis.

## Results

**Fig. 1.**
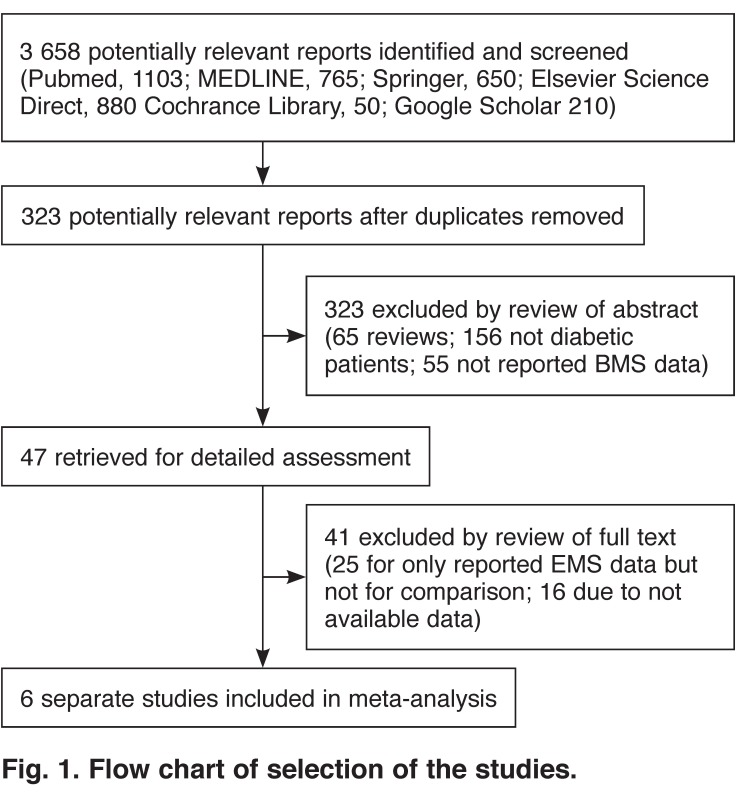
Flow chart of selection of the studies.

As shown in Fig. 1, among 3 658 articles potentially relevant to the search terms (PubMed: 1 103; MEDLINE: 765; Springer: 650; Elsevier Science Direct: 880; Cochrane Library: 50; Google Scholar: 210), 323 potentially relevant studies were selected after the duplicates were removed. When the abstracts were screened, 276 were excluded (65 were review articles, 156 were not diabetic patients, 55 did not report on BMS data). Among the remaining 47, another 41 were excluded (25 only reported on BMS data without comparisons, 16 were excluded due to unavailable data). Finally, six studies were included in this meta-analysis.

The characteristics of the included studies are presented in Table 1. These six studies were conducted from 2002 to 2006 and published between 2005 and 2008, three in Europeans, two in Americans, and one in Asians and Americans. A total of 1 259 CAD subjects with diabetes (SES 614 and BMS 645) were included, with an average age of 65 years. The sample sizes ranged from 83 to 458, and the studies were RCTs and non-RCTs.

**Table 1:** Characteristics Of Studies Included In The Meta-Analysis

						*SES group*		*BMS group*	
*Study*	*Study year*	*Country*	*Ethnicity*	*Study method*	*Follow up (years)*	*Sample size*	*Age (years)*	*Sample size*	*Age (years)*
Aoki J, *et al.*	2002–2003	Netherlands	European	Non-RCT	1	112	63 ± 10	118	64 ± 11
Jimenez-Quevedo P, *et al.*	2003	United States	America	RCT	1	80	65.4 ± 8	80	67.9 ± 9
Baumgart D, *et al.*	2002–2004	Germany	European	RCT	1	94	66 ± 9	96	66 ± 10
Daemen J, *et al.*	2002–2003	United States	America	Non-RCT	1	206	62.0 ± 10	252	62.7 ± 10
Chan C, *et al.*	2002–2004	United States and Asia	America and Asian	RCT	1	54	58.7 ± 9.7	29	62.5 ± 10.3
Maresta A, *et al.*	2004–2006	Italy	European	RCT	1	68	71 ± 9	70	69 ± 9

The efficacy of SES versus BMS is presented in Table 2. As shown, the pooled OR was 0.42 (95% CI: 0.24–0.74, *p* < 0.01) for SES versus BMS. This suggests that, after the data had been pooled, SES were more effective than BMS in CAD patients with diabetes. However, there was publication bias (*t* = –4.19, *p* < 0.05).

**Table 2:** Pooled Odds Ratio For The SES Versus The BMS Group

		*Random model*			*Test of heterogeneity*			*Egger’s test for publication bias*		
*Subgroups*	*No. of studies*	*OR (95% CI)*	*Z*	p* value*	*Q*	p*-value*	*I2 (%)*	*t*	p*-value*
Overall effects	6	0.42 (0.24–0.74)	3.00	< 0.01	20.14	< 0.01	75.2	–4.19	0.014
Sample size ≤ 90	3	0.28 (0.16–0.48)	4.60	< 0.01	2.39	0.303	16.3	–3.66	0.62
Sample size > 90	3	0.61 (0.31–1.21)	1.42	0.15	8.70	0.013	77.0	–9.26	0.20
RCT	4	0.28 (0.19–0.42)	6.14	< 0.01	2.40	0.495	0.0	–2.36	0.531
Non-RCT	2	0.87 (0.61–1.24)	0.76	0.446	0.92	0.338	0.0	–5.29	–
European	3	0.45 (0.27–0.77)	2.95	< 0.01	3.71	0.156	46.1	–7.98	0.46
American and Asian	3	0.37 (0.11–1.27)	1.58	0.115	15.55	< 0.01	87.1	–5.92	0.23

**Fig. 2.**
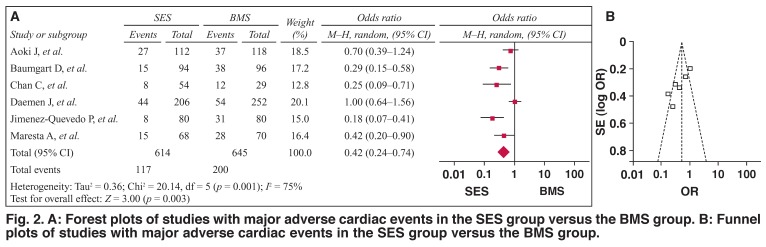
A: Forest plots of studies with major adverse cardiac events in the SES group versus the BMS group. B: Funnel plots of studies with major adverse cardiac events in the SES group versus the BMS group.

As shown in Fig. 2A, the pooled OR was 0.42 (95% CI: 0.24–0.74, *p* < 0.01) for overall events, suggesting that SES had a better outcome compared with BMS, with a greater reduction in risk for major cardiac events. However, there were heterogeneities between the studies (*Q*^2^ = 20.14, *I*^2^ = 75.0%, *p* < 0.1) and publication bias, as shown in Fig. 2B (asymmetric funnel plot). This was further confirmed with Egger’s linear regression test, shown in Table 2 (*t* = –4.19, *p* < 0.05).

**Fig. 3.**
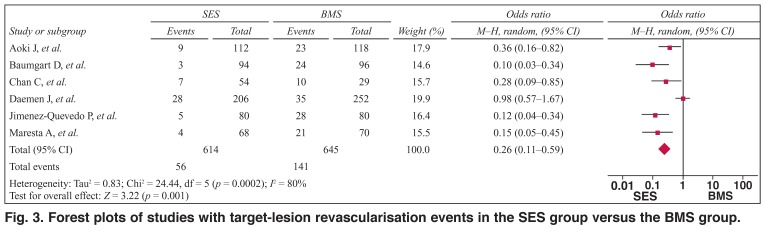
Forest plots of studies with target-lesion revascularisation events in the SES group versus the BMS group.

As shown in Fig. 3, the pooled OR was 0.26 (95% CI: 0.11–0.59, *p* < 0.01) for SES versus BMS, suggesting that SES had a better revascularisation rate for target lesions compared with BMS. However, there were heterogeneities between the studies (*Q*^2^ = 24.44, *I*^2^ = 80.0%, *p* < 0.1) and publication bias (*t* = –6.44, *p* < 0.05).

**Fig. 4.**
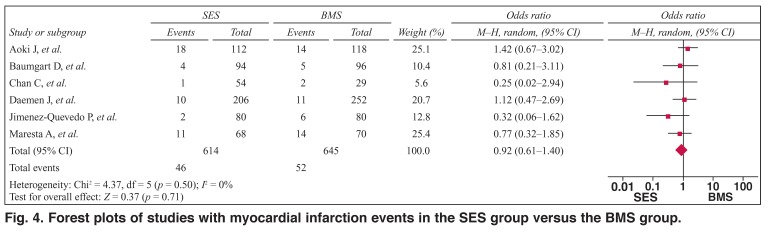
Forest plots of studies with myocardial infarction events in the SES group versus the BMS group.

As shown in Fig. 4, the pooled OR was 0.92 (95% CI: 0.61–1.40, *p* > 0.05) for SES versus BMS, suggesting that the overall risk for myocardial infarction was not significantly different between these two groups. There was no heterogeneity between the studies (*Q*^2^ = 4.37, *I*^2^ = 0%, *p* > 0.1) but there was publication bias (*t* = –3.44, *p* < 0.05).

**Fig. 5.**
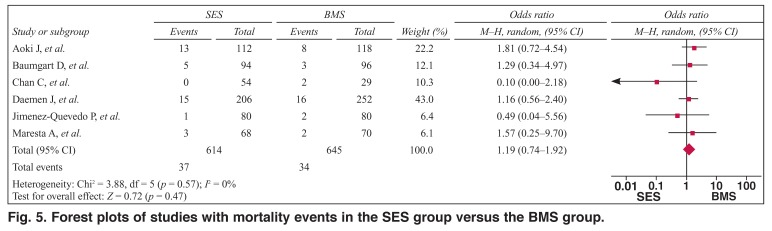
Forest plots of studies with mortality events in the SES group versus the BMS group.

As shown in Fig. 5, the pooled OR was 1.19 (95% CI: 0.74–1.92, *p* > 0.05) for SES versus BMS, suggesting that the overall risk of mortality was not significantly different between the groups. There was no publication bias (*t* = –1.69, *P* > 0.05) or heterogeneities between the studies (*Q*^2^ = 3.88, *I*^2^ = 0.0%, *p* > 0.1).

**Fig. 6.**
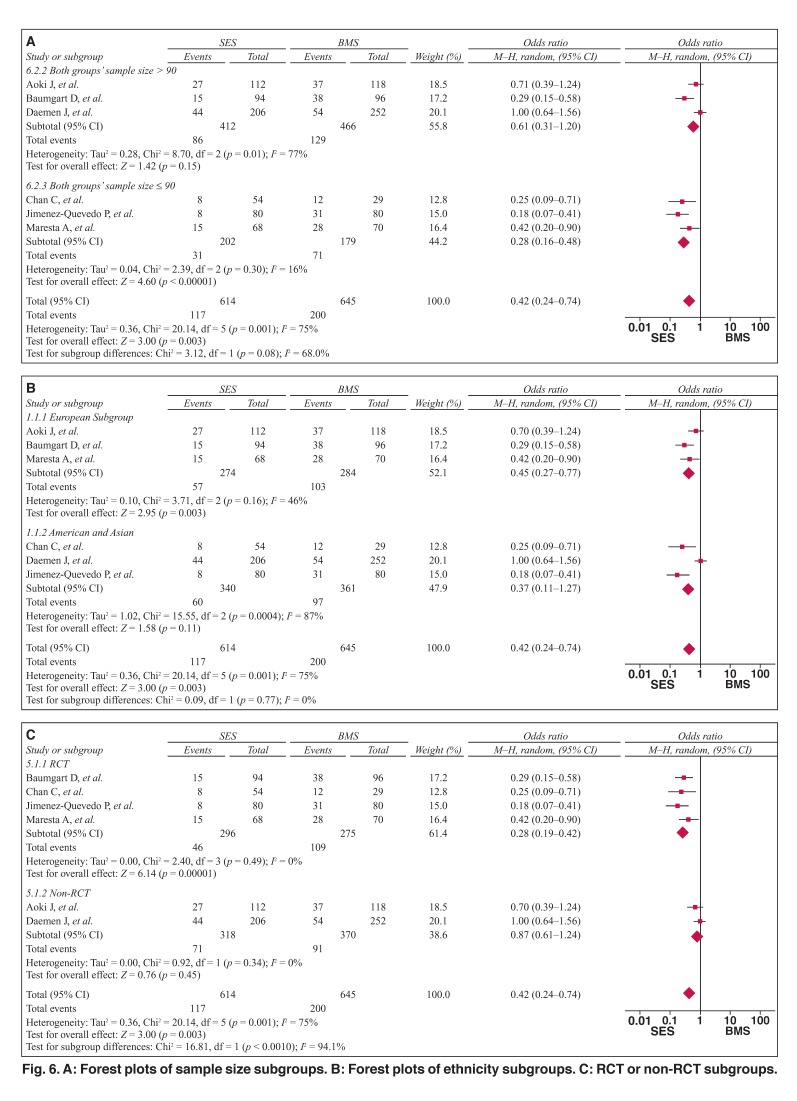
A: Forest plots of sample size subgroups. B: Forest plots of ethnicity subgroups. C: RCT or non-RCT subgroups.

Subgroup analyses were stratified by sample size, subjects’ geographical area and study method. As shown in Table 2 and Figure 6A–C, the pooled OR was 0.28 (95% CI: 0.16–0.48, *p* < 0.01, Fig. 6A) for SES versus BMS in studies whose sample size was above 90, with heterogeneities between the studies (Q2 = 8.7, *I*^2^ = 77%, *p* < 0.1). The pooled OR was 0.61 (95% CI: 0.31–1.21, *p* > 0.05, Fig. 6A) in studies whose sample size was 90 or less, without heterogeneities between the studies (*Q*^2^ = 2.39, *I*^2^ = 16%, *p* > 0.1).

The pooled OR was 0.45 (95% CI = 0.27–0.77, *p* < 0.01, Fig. 6B) in studies whose subjects were European, without heterogeneities between the studies (*Q*^2^ = 3.71, *I*^2^ = 46%, *p* > 0.1). The pooled OR was 0.37 (95% CI: 0.11–1.27, *p* > 0.05, Fig. 6B) in studies whose subjects were American and Asian, with heterogeneities between the studies (*Q*^2^ = 15.55, *I*^2^ = 87%, *p* < 0.1).

The pooled OR was 0.28 (95% CI: 0.19–0.42, *p* < 0.01, Fig. 6C) in studies whose study method was RCT, without heterogeneities between the studies (*Q*^2^ = 2.4, *I*^2^ = 0%, *p* > 0.1). The pooled OR was 0.87 (95% CI: 0.61–1.24, *p* > 0.05, Fig. 6C) in studies whose method of study was non-RCT, without heterogeneities between the studies (*Q*^2^ = 0.92, *I*^2^ = 0%, *p* > 0.1).

By removing one study at a time, a sensitivity analysis was performed and the model was rerun to determine the effect on each estimate. It showed that the above meta-analysis estimates did not change significantly after removal of each study, implying that these results were statistically reliable.

## Discussion

A growing number of studies has shown the efficacy and safety of SES versus BMS for treating CAD patients with diabetes,^9^,^29^ but the outcome has been controversial. In this analysis, we retrieved six studies, which included 1 259 CAD subjects with diabetes, and performed a meta-analysis. It showed that the SES group had a significant reduction in major adverse cardiac events, as well as target-lesion revascularisations, compared with the BMS group. There was no significant difference for myocardial infarction or mortality.

These results are consistent with a recent study that suggested a significant reduction in target-vessel revascularisations with SES, but with similar mortality rates.^9^ Unlike this study, in which the incidence of myocardial infarction was higher, our analysis showed no difference for myocardial infarctions between the groups.

Another recent study conducted in Europeans confirmed the efficacy of SES compared with BMS, along with comparable mortality rates and myocardial infarctions,^11^ which further proved the validity of our analysis. The efficacy and safety of SES have been receiving more and more supportive reports.^30^-^33^ The uniqueness of our analysis and findings is that it proved the efficacy and safety of SES in CAD patients with diabetes.

Heterogeneity is one major concern with regard to the validity of meta-analyses.^26^,^34^ Non-homogeneous data can easily give misleading results. In our study, the Q and I2 statistics were performed to test heterogeneity. For all samples, there was significant heterogeneity for major adverse cardiac events in the SES and BMS groups.

We further conducted subgroup analysis according to sample size, ethnicity and study method. It demonstrated that in the studies where sample size was ≤ 90, method was a RCT and population was European, the overall major cardiac events were significantly different between the SES and BMS groups. Heterogeneity between the studies was decreased after stratifying the samples. No significant heterogeneity was observed with RCTs, suggesting an RCT is important for good results. More high-quality RCTs are therefore warranted.

Another concern for meta-analyses is publication bias, due to selection of the studies included. In this study, using funnel plots and Egger’s test,^28^,^35^,^36^ we found publication bias for overall major cardiac events, target-lesion revascularisations and myocardial infarction, but not for overall mortality. Furthermore, the sensitivity analysis confirmed there was no change if one study was removed at a time. Although more studies would have produced better results, overall, our results were statistically reliable.

## Conclusion

This meta-analysis suggested that, compared with BMS, SES are more effective and safer for reducing major cardiac events in CAD patients with diabetes. This may indicate the direction for future trials and clinical implementation.
